# Geographical distribution of invasive meningococcal disease and carriage: A spatial analysis

**DOI:** 10.1017/S0950268824000116

**Published:** 2024-01-18

**Authors:** Adriana Milazzo, Mark McMillan, Lynne Giles, Kira Page, Louise Flood, Helen Marshall

**Affiliations:** 1 School of Public Health, The University of Adelaide, Adelaide, Australia; 2 Vaccinology and Immunology Research Trials Unit, Women’s and Children’s Health Network, Adelaide, Australia; 3 Robinson Research Institute, The University of Adelaide, Adelaide, Australia; 4 Adelaide Medical School, The University of Adelaide, Adelaide, Australia; 5 Australian Centre for Housing Research, Hugo Centre for Population and Migration Studies, The University of Adelaide, Adelaide, Australia; 6 Communicable Disease Control Branch, Department for Health and Wellbeing, Government of South Australia, Adelaide, Australia

**Keywords:** meningococcal disease, meningococcal carriage, geographical distribution, Neisseria meningitidis, Vaccination

## Abstract

Little information exists concerning the spatial relationship between invasive meningococcal disease (IMD) cases and *Neisseria meningitidis (N. meningitidis)* carriage. The aim of this study was to examine whether there is a relationship between IMD and asymptomatic oropharyngeal carriage of meningococci by spatial analysis to identify the distribution and patterns of cases and carriage in South Australia (SA). Carriage data geocoded to participants’ residential addresses and meningococcal case notifications using Postal Area (POA) centroids were used to analyse spatial distribution by disease- and non-disease-associated genogroups, as well as overall from 2017 to 2020. The majority of IMD cases were genogroup B with the overall highest incidence of cases reported in infants, young children, and adolescents. We found no clear spatial association between *N. meningitidis* carriage and IMD cases. However, analyses using carriage and case genogroups showed differences in the spatial distribution between metropolitan and regional areas. Regional areas had a higher rate of IMD cases and carriage prevalence. While no clear relationship between cases and carriage was evident in the spatial analysis, the higher rates of both carriage and disease in regional areas highlight the need to maintain high vaccine coverage outside of the well-resourced metropolitan area.

## Introduction

Invasive meningococcal disease (IMD), caused by *Neisseria meningitidis (N. meningitidis)*, affects populations worldwide, particularly young children, adolescents, and young adults, with ensuing morbidity and mortality. Globally, the estimated incidence of IMD during 2010–2019 pooled across 30 countries ranged from 0.0 to 10.2 per 100,000 population [1]. Although an uncommon disease, the sequelae associated with infection can be severe, leading to lifelong disability or death. Of the 12 *N. meningitidis* genogroups, A, B, C, W, X, and Y are associated with causing invasive disease. In a pooled analysis (across 2000–2017) including studies from many countries, 48.5% of cases were caused by genogroup B [2].

In Australia, genogroup B meningococci were responsible for 50% of all IMD cases notified in 2019, highest in children aged <5 years (25%), adolescents aged 15–19 years (13%), and young adults aged 20–24 years (11%) [3, 4]. Compared to other Australian states and territories, the notification rate for IMD in 2018 was the third highest in the state of South Australia (SA), the location for this study, with two cases per 100,000 population, and in 2019, it was the second highest at 1.5 cases per 100,000 population [3]. One-third of invasive meningococcal B cases in adolescents reported nationally were in SA [4].

Pharyngeal carriage of *N. meningitidis* is necessary for IMD to occur. The prevalence of asymptomatic *N. meningitidis* carriage is around 10% [5]. Individuals may be chronic carriers, with carriage lasting several months, or intermittent or transient carriers. Carriage is higher in adolescents and young adults; consequently, individuals in this age group are important transmitters of the disease [6, 7].

While there have been a number of meningococcal carriage studies concerning prevalence among age-specific groups [7] and high-risk settings [8], as well as the impact of vaccine on carriage or disease [9–11], there is limited evidence on the transmission dynamics of *N. meningitidis* carriage and IMD with few studies assessing the relationship between carriage prevalence and disease incidence. A carriage study of adolescents in the United Kingdom found differences in IMD incidence according to meningococcal carriage prevalence and lower carriage prevalence during periods of low IMD incidence [12]. Similarly, carriage rates were positively correlated with IMD incidence, and high carriage prevalence was associated with IMD outbreaks in studies from China and northern Ghana [13, 14], but lower rates of carriage were reported during a meningococcal A outbreak in the African meningitis belt [15]. Given the paucity of studies that have considered this and the inconsistency in findings, whether disease incidence is related to carriage prevalence should be further explored. One way to address this gap is to examine the spatial distribution of IMD cases and carriage as it may provide new insights into understanding the links between them. Previous studies have used spatial analysis to identify clustering of IMD incidence and outbreaks, mainly in Europe [16–18] and sub-Saharan Africa [19–23], with the aim of understanding transmission patterns geographically to enable improved disease prevention and control. However, few studies have used spatial analysis to jointly examine IMD incidence and carriage prevalence, nor have they spatially analysed cases and carriage by *N. meningitidis* genogroup.

From 2017 to 2020, a large randomized controlled trial (RCT) (‘B Part of It’) studying the impact of the meningococcal B (4CMenB) vaccine on the carriage of *N. meningitidis* among secondary school students and school leavers was undertaken across SA [10, 24]. The aim of the present study was to spatially relate the incidence of IMD cases to *N. meningitidis* carriage prevalence (from ‘B Part of It’), both overall and by genogroup, from 2017 to 2020, to identify the distribution and patterns of cases and carriage in SA. Improved understanding of meningococcal carriage will give an indication of how widespread it is and may also assist in further understanding the relationship with the disease by establishing whether the geographical distribution of carriage is similar to that of IMD.

## Methods

**Figure 1.**
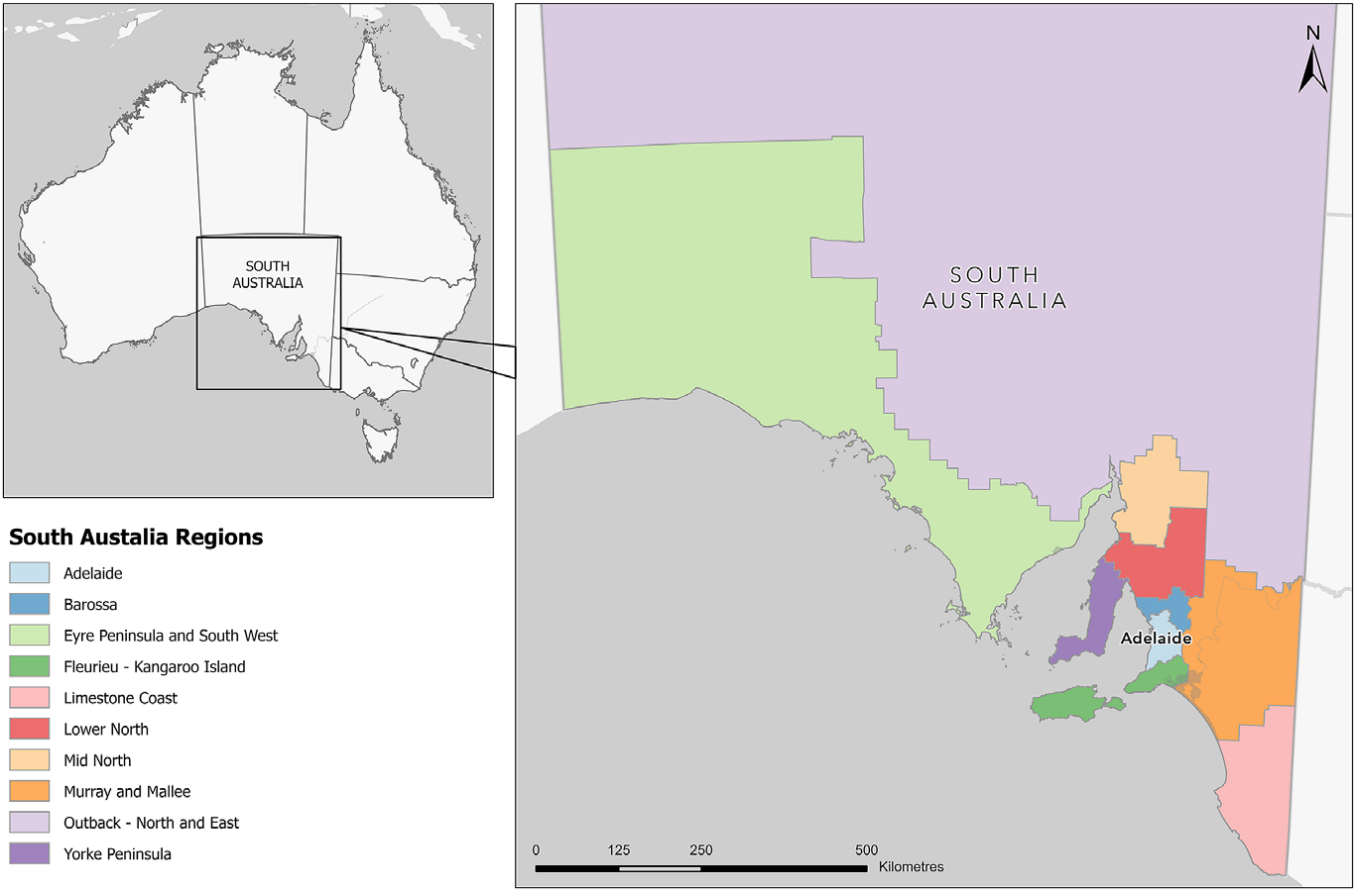
Map of South Australia by region.

The setting for this study was SA, a state in the southern central part of Australia (Figure 1). In 2021, the estimated population of SA was 1.77 million people, of which 1.32 million (75%) lived in the capital city of Adelaide (https://plan.sa.gov.au/state_snapshot/population). There were nine rural regions outside the Adelaide Statistical Division Boundary (the metropolitan area) (Figure 1).

## Data sources

IMD is a notifiable disease in Australia. Daily laboratory-confirmed IMD cases for individuals residing in SA who were notified to the Communicable Disease Control Branch (CDCB), South Australian Department for Health and Wellbeing, between 1 January 2017 and 31 December 2020 were obtained from the CDCB notifiable disease surveillance system. The date of onset of illness and demographic characteristics were extracted along with the meningococcal genogroup identified for each case.

This study used *N. meningitidis* carriage prevalence from the ‘B Part of It’ (ClinicalTrials.gov number, NCT03089086) RCT spanning 2017 to 2020. Students aged 15–18 years enrolled across 237 South Australian secondary schools participated in the study, along with a second group of eligible participants aged 17–25, who completed their school education in the year prior to enrolling in the study [9, 24]. Participant demographic characteristics included residential address and school location. Oropharyngeal swabs were collected, and a polymerase chain reaction (PCR) test was undertaken to screen for meningococcal deoxyribonucleic acid (DNA). PCR testing identified *Por*A (encoding porin protein A) and disease-associated *N. meningitidis* due to genogroup A, B, C, W, X, or Y [9, 25]. Non-groupable carriage was defined as failure to detect genogroup A, B, C, W, X, or Y in those with *Por*A detected.

## Spatial analysis

### Invasive meningococcal disease notification data

We analysed the spatial pattern of IMD notifications from 2017 to 2020 overall and by genogroup. Cases were aggregated by postcode of residence and mapped to the Australian Bureau of Statistics (ABS) 2016 Postal Area (POA) by centroids [26]. Taken as the primary spatial unit of analysis, POA is an ABS approximation of postcodes, with postcode centroids showing the approximate centre that the postcode applies to. Postcodes usually apply to more than one suburb and, in some instances, span state/territory borders. Taking the postcode of IMD cases to be centrally located also minimized the potential for identification of cases at the individual level.

### *N. meningitidis* carriage

To analyse the spatial pattern of *N. meningitidis* carriage in total and by genogroup, pooled data from the carriage prevalence study across 2017–2020 were geocoded to participants’ residential addresses using Esri’s ArcGIS World Geocoding Service [27]. Participants’ South Australian residential addresses were selected for density mapping to avoid a clustering of carriage detections at the school locations, which would obscure patterns of distribution.

The accuracy of geocoded addresses can vary, and each geocoded address was classified as having a high or low accuracy match. The accuracy of geocoding depends on the degree of closeness of an address to its actual value. High geocoding accuracy records included those where geocoding was based on House Match, Estimated House Match, Manual Estimated House Match, or Street Centroid. Records with low geocoding accuracy were those where geocoding was to Suburb Match, Manual Suburb Match, Postcode Match, School/Business Address Match, or No Match. Records with low geocoding accuracy, records geocoded outside of SA, or those with missing genogroup were excluded from subsequent analyses.

An analysis to calculate the density of individual carriage data that fall within an area was performed on the included geocoded records to identify patterns of any *N. meningitidis* carriage detected and patterns of disease-associated meningococci – genogroup A, B, C, W, X, or Y detected in oropharyngeal swabs. In addition, we calculated the density for all participants sampled. As the Adelaide metropolitan area contained the highest concentration of records, point density analyses were limited to the 2011 Adelaide Statistical Division Boundary [28]. To illustrate and explore in detail the geospatial distribution of cases and carriage across SA, an interactive web mapping application has been developed and is accessible at (http://spatialonline.com.au/meningococcalspatialmap).

The application includes the map of SA with two layers: the first shows the number of IMD cases aggregated to local government area (LGA) centroids, and the second shows the distribution of all *N. meningitidis* carriage across SA for the entire study period aggregated to LGA boundaries. LGAs are gazetted boundaries defined by jurisdictional governments and are larger spatial units, thus reducing the potential for identifiability of cases or carriage. Cases and carriage can be overlapped, and the distribution can be examined at a closer extent (not by street address) by selecting the plus (+) or minus (−) zoom functions on the map. Areas of interest can be selected to reveal a popup window with details on the number of cases by genogroup and meningococcal carriage genogroup (including non-groupable meningococci) along with the population data for each LGA.

We calculated the overall notification rate (per 100,000 population) for IMD cases across 2017–2020 based on the postcode assigned to an LGA. Population data for each LGA were obtained from the ABS 2016 Census LGAs (https://abs.gov.au/census/find-census-data/quickstats/2016/CED411). Disease-associated carriage prevalence was calculated based on the number of swabs collected for each LGA during the study period. Overall IMD notification rate and disease-associated carriage prevalence were calculated for combined metropolitan and combined regional LGAs (in LGAs where cases or carriage were detected). A test of proportions compared differences in the rate and prevalence between the two regions. Wald 95% confidence interval (CI) was calculated assuming a binomial distribution [29]. Pearson’s correlation coefficient was used to examine the relationship between IMD incidence and disease-associated carriage prevalence reported in LGAs and was calculated separately for metropolitan and regional areas. All spatial analyses were performed in the GDA 2020 SA Lambert coordinate system. ArcGIS Pro software (Esri version 2.9.0) was used for all spatial analyses. Spatial data exported to the web map were reprojected into WGS 1984 Web Mercator (auxiliary sphere) – the default web geographic coordinate system (GIS) (https://doc.arcgis.com/en/arcgis-online/reference/faq.htm).

### Ethics

All data analysed were non-identifiable, and ethics approval was given by the Human Research Ethics Committee of the South Australian Department for Health and Wellbeing (HREC/19/SAH/8) and the Women’s and Children’s Health Network Human Research Ethics (2021/HRE00179).

## Results

### Invasive meningococcal disease notification data

**Figure 2.**
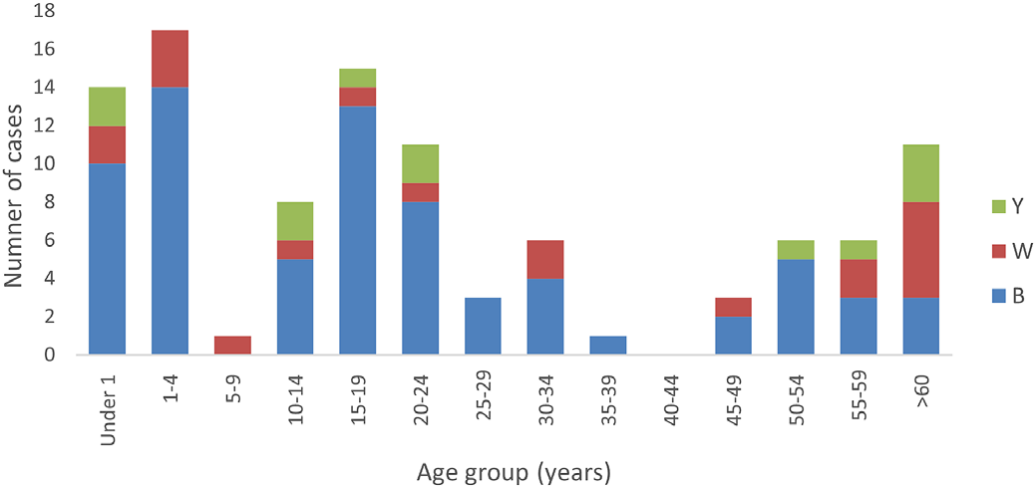
Number of meningococcal cases notified in South Australia by age group and genogroup, 2017–2020.

There were 102 IMD notifications reported in the four-year study period. The number of cases ranged from 5 to 39 cases per year, with the minimum annual count reported during the first year of the COVID-19 pandemic. Of the 102 cases, 71 (69.6%) were further characterized as genogroup B, 19 (18.6%) cases as genogroup W, and 12 (11.8%) cases as genogroup Y. There were no IMD genogroup A, C, or X cases notified during this period. The highest number of cases overall was reported in infants and young children aged 0–4 years (n = 31, 30.4%), followed by adolescents aged 15–19 years (n = 15, 14.7%) (Figure 2).

This pattern was similar for the 71 IMD genogroup B cases, with the highest proportion reported in the 0- to 4-year age group (n = 24, 33.8%) and those aged 15–19 years (n = 13, 18.3%). By contrast, there was a higher proportion of IMD genogroup W cases in those aged over 60 years (n = 5 of 19, 26.3%) and among children in the 0- to 4-year age group (n = 5, 26.3%). For IMD genogroup Y, the number of cases in infants, adolescents, and young adults was evenly distributed with slightly more cases in the over-60-year-old age cohort. More than two-thirds of cases were reported in the under-25-year-old group compared with those older than 25 years of age. In keeping with the residential distribution of the population of SA, the majority of IMD cases (n = 72, 70.6%) lived in metropolitan Adelaide, with the remaining cases in regional areas. A similar pattern was observed for genogroups B and Y. The distribution differed somewhat for genogroup W cases, with 11 (57.9%) residing in Adelaide and eight (42.1%) in regional areas.

### *N. meningitidis* carriage

**Figure 3.**
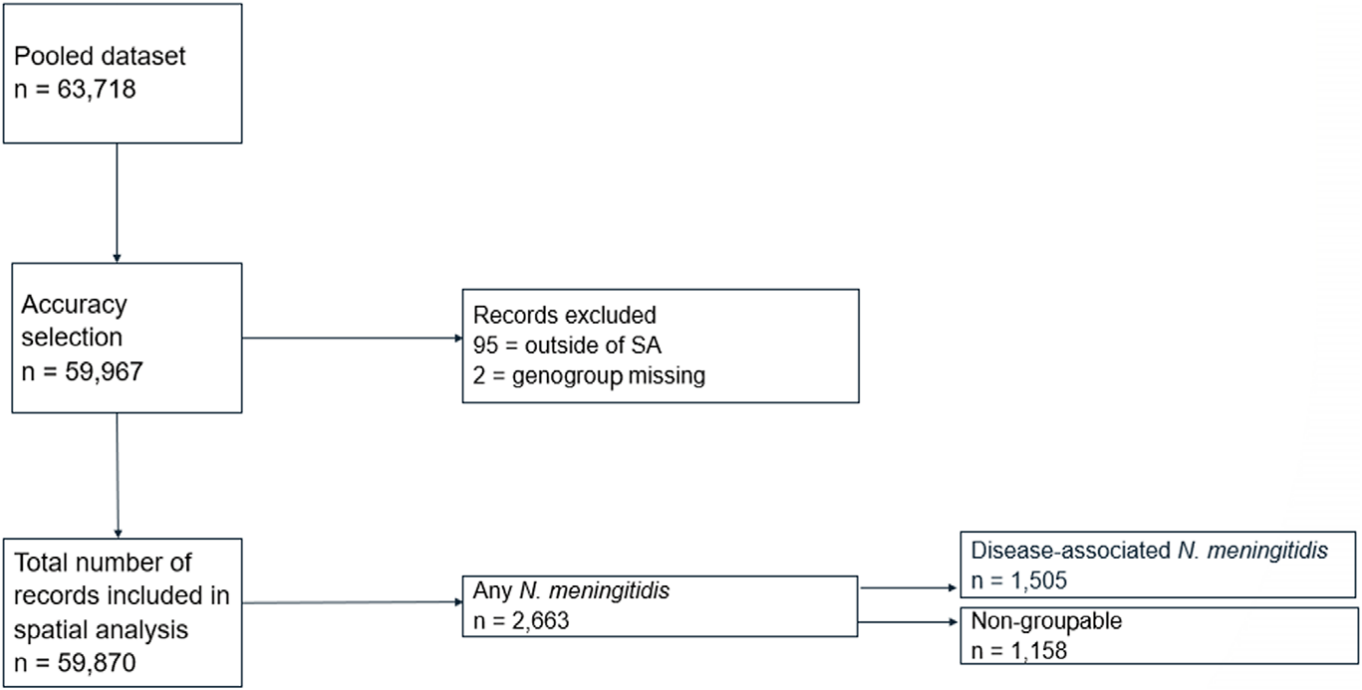
Exclusion and inclusion of disease-associated *Neisseria meningitidis* carriage per record, 2017–2020.

A total of 63,718 oropharyngeal swabs were collected, and PCR was undertaken to detect disease and non-disease-associated *N. meningitidis* from the pooled data set of school students and school leavers. Most swabs were taken from RCT participants who had repeat testing in 2017 and 2018. However, in 2019–2020, the RCT participants represented only 74% of the total swabs. Excluding addresses with low geocoding accuracies resulted in a subset of the pooled data of 59,967 records. Ninety-five of these records were excluded as they were outside of SA, and for two records, the genogroup was missing despite the detection of *PorA.* Following the removal of these records, 59,870 records were included for spatial analysis (and for calculating the carriage prevalence rate). Carriage of any *N. meningitidis* was detected in 2,663 oropharyngeal swabs, resulting in a carriage prevalence of 4.44% (95% CI 4.28%–4.61%), and disease-associated carriage was detected in 1,505 swabs, giving a disease-associated carriage prevalence of 2.51% (95% CI 2.38%–2.64%). The non-groupable genogroup was reported in 1,158 oropharyngeal swabs (Figure 3). Multiple genogroups were detected in 42 (2.7%) swabs, totalling 1,547 genogroups identified from the 1,505 swabs. Meningococcal genogroup B accounted for the highest number of swabs indicating disease-associated carriage. There was no carriage of genogroup A reported (Table 1).

**Table 1.** Carriage of disease-associated genogroup, all of South Australia, 2017–2020

Carriage	Carriage of disease-associated genogroup N = 1,505^a^ (%)
Carriage of genogroup B	752 (48.6%)
Carriage of genogroup Y	596 (38.5%)
Carriage of genogroup W	112 (7.2%)
Carriage of genogroup C	63 (4.1%)
Carriage of genogroup X	24 (1.6%)
Total^b^	1,547

a n = 1,505 individual oropharyngeal swabs.

b total counts sum to 1,547 due to multiple genogroups detected on some swabs. The denominator used to calculate the proportion of each of the disease-associated genogroups was 1,547^#^.

### Geographical distribution

#### Disease-associated carriage in South Australia

**Figure 4.**
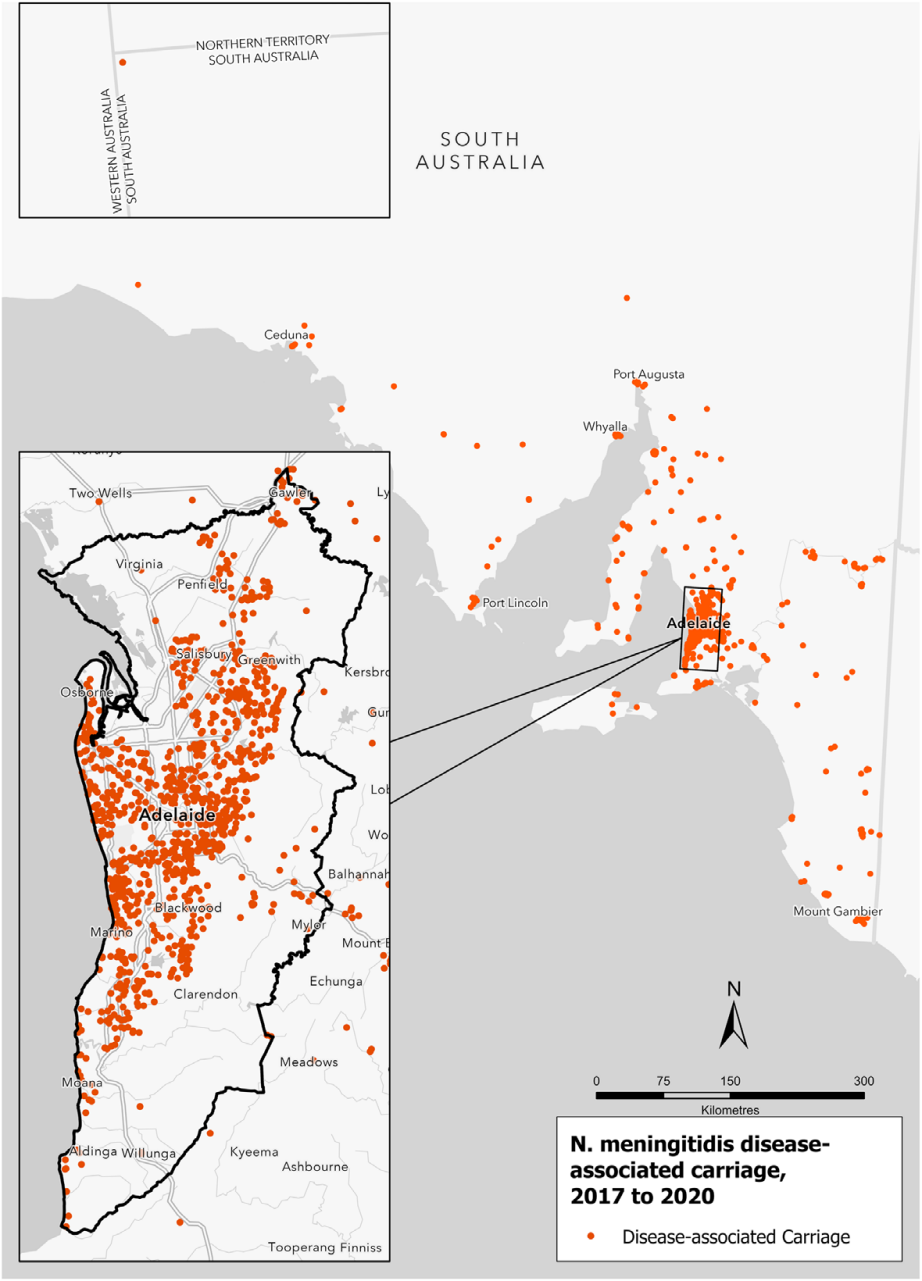
*Neisseria meningitidis* disease-associated carriage, by all genogroups, Adelaide and South Australia, 2017 to 2020.

The distribution of *N. meningitidis* disease-associated carriage was spread across regional SA, with the detection of carriage observed across the Eyre Peninsula (Ceduna and Port Lincoln in the west), Yorke Peninsula, Mid North (Port Augusta in the north), and Limestone Coast (Mt Gambier in the south-east). The inset shows carriage distribution appeared randomly spread across the Adelaide metropolitan area with no discernible pattern (Figure 4).

#### Case and carriage density in Adelaide

**Figure 5.**
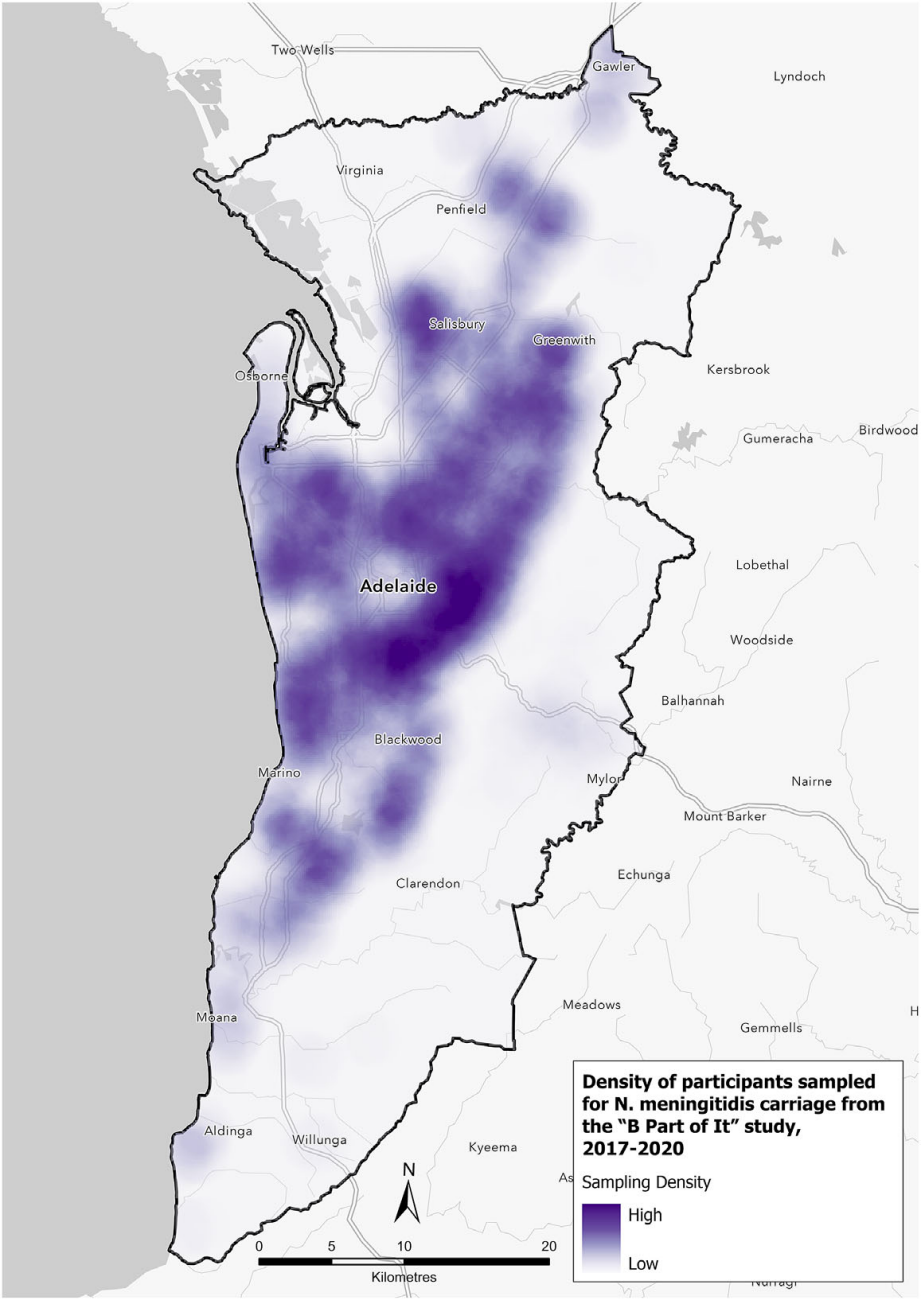
Density of participants sampled, Adelaide, 2017 to 2020.

**Figure 6.**
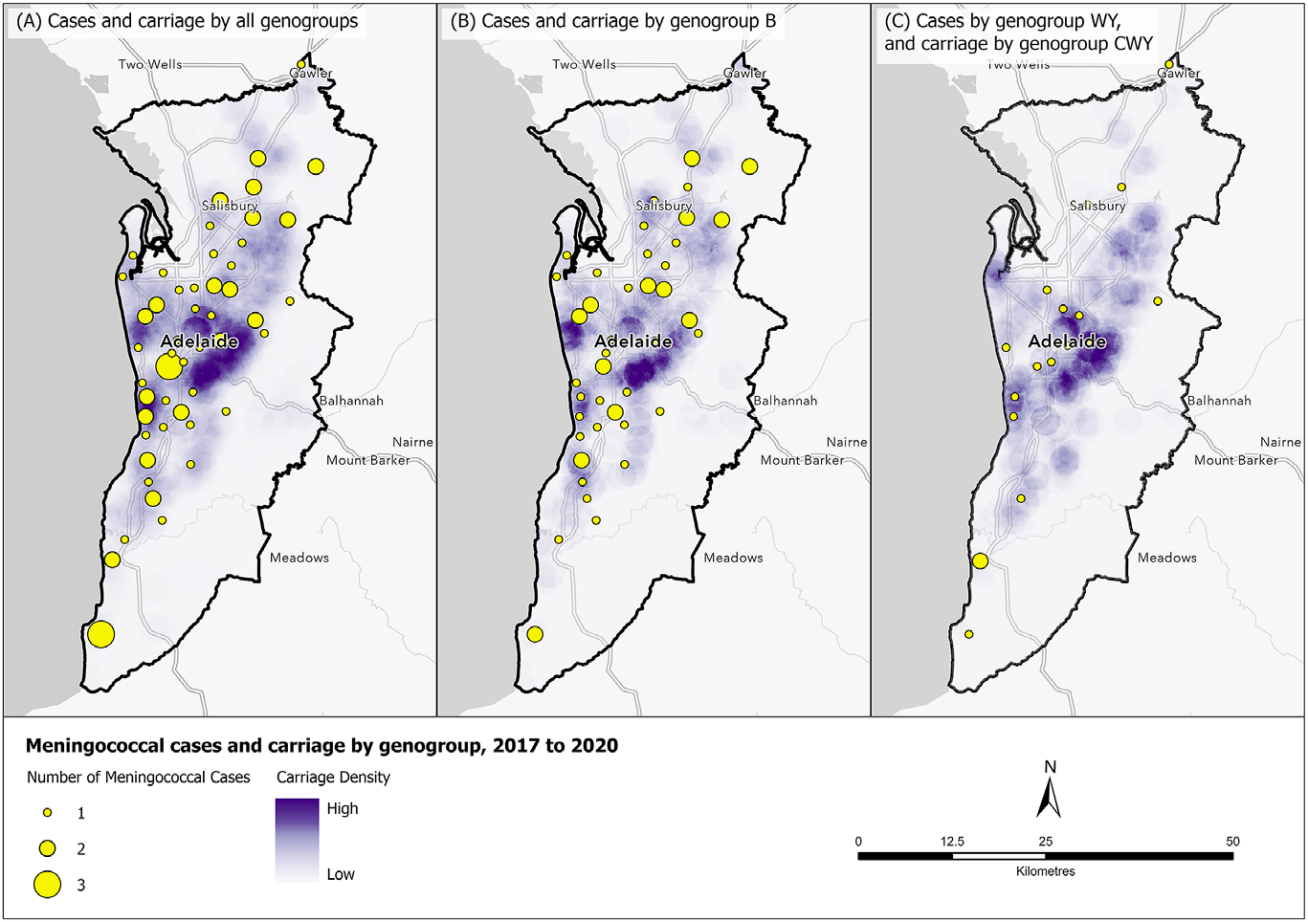
Number of meningococcal cases and *N. meningitidis* carriage by (a) all genogroups; (b) genogroup B; (c) genogroups W and Y (cases) and C, W, and Y (carriage), Adelaide, 2017 to 2020.

Density sampling of participants in the Adelaide Statistical Division Boundary (metropolitan area) was higher across the east and north-west of Adelaide (Figure 5). Figure 6a shows the distribution of meningococcal carriage and IMD cases within the metropolitan area. A higher density of any *N. meningitidis* carriage (for all genogroups and non-groupable) was identified in the north-east and south of Adelaide, while the distribution of IMD cases was spread throughout Adelaide with no discernible pattern observed between carriage prevalence and case incidence. A higher density of *N. meningitidis* carriage genogroup B was observed in the east and west of Adelaide with counts of IMD cases spread throughout metropolitan Adelaide (Figure 6b). Carriage density for genogroups C, W, and Y was higher in the east and north of Adelaide, but IMD cases by genogroups W and Y were sparse (Figure 6c).

#### IMD notification rates by local government area

The five LGAs with the highest rate of IMD cases observed in the Adelaide metropolitan area and regional SA are reported in Table 2. Of the 18 LGAs in the metropolitan area, 15 (83.3%) recorded IMD case(s). IMD notification rates per 100,000 population across the four-year study period were highest in the Holdfast Bay LGA, south of Adelaide (14.14 cases per 100,000 population), followed by notification rates in the east and west of Adelaide.

**Table 2.** Local government areas (LGAs) with the highest IMD notification rate, and IMD notification rates for combined LGAs, per 100,000 population, Adelaide (metropolitan) and regional South Australia, 2017–2020

Metropolitan				
LGA	Adelaide Statistical Division Boundary	Population size (2016)	IMD notification rate per 100,000 population	95% CI
Holdfast Bay	South	35,360	14.14	1.75–26.53
Walkerville	East	7,550	13.24	0–39.20
Mitcham	South	64,805	7.71	0.95–14.48
Port Adelaide Enfield	West	121,230	7.42	2.57–12.27
West Torrens	West	57,901	6.90	0.13–13.68
Combined 18 LGAs	Adelaide (metropolitan)	1,227,685	5.87	4.51–7.22

Regional				
LGA	Region within South Australia^a^	Population size (2016)	IMD notification rate per 100,000 population	95% CI
Unincorporated SA	Outback – North and East	3,524	141.88	17.61–266.16
Ceduna	Eyre Peninsula and South West	3,408	88.02	0–18.76
Mid Murray	Lower North	8,642	23.14	0–55.21
Yankalilla	Fleurieu–Kangaroo Island	5,159	19.38	0–57.37
The Coorong	Limestone Coast	5,380	18.58	0–55.01
Combined 21 LGAs	Regional South Australia	298,568	10.05	6.45–13.64

a Regions defined as per Figure 1: Map of South Australia by region.

Of the 52 LGAs in regional SA, 21 (40.4%) LGAs reported one or more IMD cases. Unincorporated *S*A (not governed by local council authority and within the Outback – North and East region) had the highest rate of cases (141.88 per 100,000 population) followed by cases in the far west and north of SA. The lower-bound 95% CI of 0 and wider 95% CI for some of the LGAs reflected the low number of cases and small population size. The IMD notification rate across 2017–2020 was highest for regional LGAs (10.05, 95% CI 6.45–13.64 per 100,000 population) compared with metropolitan LGAs (5.87, 95% CI 4.51–7.22 per 100,000 population) (Table 2). There was a difference in the IMD notification rate (difference 4.19, 95% CI 0.34–8.03 per 100,000 population, *P*-value = 0.012) with IMD cases more likely to be reported from regional SA than from metropolitan Adelaide.

#### Proportion of disease-associated carriage by local government area

Table 3 shows the five LGAs with the highest proportion of disease-associated carriage in metropolitan Adelaide and regional SA. All 18 LGAs in Adelaide reported the detection of disease-associated carriage from swabs. The proportion of disease-associated carriage (6.4%) was highest in the Adelaide LGA (centre of Adelaide) (Table 3).

**Table 3.** Local government areas (LGAs) with the highest prevalence of disease-associated carriage and prevalence of disease-associated carriage for combined LGAs, Adelaide (metropolitan) and regional South Australia, 2017–2020

Metropolitan Adelaide					
LGA	Adelaide Statistical Division Boundary	Number of swabs^a^	Number of swabs detected disease-associated carriage^a^	Prevalence of disease-associated carriage^a^	95% CI
Adelaide	Central	388	25	6.44%	4.00%–8.88%
Holdfast Bay	South	1,135	48	4.22%	3.05%–5.39%
Unley	South	1,563	53	3.39%	2.49%–4.28%
Burnside	East	2,449	80	3.26%	2.56%–3.97%
Prospect	North	785	24	3.05%	1.85%–4.26%
Combined 18 LGAs	Adelaide (metropolitan)	45,626	1,122	2.46%	2.31%–2.60%

Regional South Australia					
LGA	Region within South Australia^b^	Number of swabs^a^	Number of swabs detected disease-associated carriage^a^	Prevalence of disease-associated carriage^a^	95% CI
Wudinna	Eyre Peninsula and South West	35	5	14.28%	2.69%–25.87%
Unincorporated SA	Outback – North and East	48	5	10.41%	1.77%–29.05%
Karoonda East Murray	Murray and Mallee	34	3	8.82%	0%–18.35%
Southern Mallee	Murray and Mallee	45	3	6.66%	0%–13.95%
Anangu Pitjantjatjara	Outback – North and East	18	1	5.55%	0%–16.13%
Combined 53 LGAs	Regional South Australia	15,868	423	2.67%	2.41%–2.91%

a Swabs were taken from school-aged students and school leavers residing in metropolitan and regional areas who participated in the ‘B Part of It’ study from 2017 to 2020.

b Regions defined as per Figure 1: Map of South Australia by region.

Of the 52 LGAs in regional SA, 46 (88.5%) reported the detection of disease-associated carriage from swabs. A high proportion of disease-associated carriage was observed in multiple regional LGAs, including the Wudinna area (14.3%) located in the Eyre Peninsula (north-west), Outback – North and East, and Murray Mallee Region – north of SA. Some estimates are large given the small population sizes of LGAs in regional areas. The prevalence of disease-associated carriage across 2017–2020 was slightly higher for regional LGAs (2.67%, 95% CI 2.41%–2.91%) compared with metropolitan LGAs (2.46%, 95% CI 2.31%–2.60%) (Table 3). There was a minimal difference in disease-associated carriage prevalence (difference −0.21%, 95% CI −0.49%–0.08%, *P*-value = 0.152) between the regional and metropolitan areas.

There was negligible correlation (r = 0.09) between IMD incidence and prevalence of disease-associated carriage in metropolitan Adelaide, while IMD and disease-associated carriage in regional SA were weakly correlated (r = 0.36).

## Discussion

Our ecological study used aggregated data to relate the geographical distribution of laboratory-confirmed IMD cases and adolescent carriage prevalence over a four-year period. In this study, IMD genogroup B meningococci were reported in the majority of cases with genogroups A, C, and X not reported. Our results are consistent with other studies outside of the meningitis belt showing that IMD genogroup B meningococci are a predominant cause of cases internationally [1, 2, 30] and in Australia, with young infants and children, and adolescents significantly impacted [3, 4]. Australia’s meningococcal vaccination programme has targeted these age groups at high risk of IMD disease [31]. The MenACWY conjugate vaccine introduced in Australia in 2018 providing coverage for infants and adolescents is likely to be a contributing factor to the absence of IMD genogroups A and C during this study period [32]. SA was also the first state to provide a funded, state-based meningococcal B vaccination programme, commencing in 2018 for infants and in 2019 for adolescents, which has also contributed to lowered incidence of genogroup B cases [4, 33]. Comparable with the low number of IMD cases notified during the study period, the prevalence of any *N. meningitidis* carriage for this study population was 4.4%, and for disease-associated carriage, the prevalence was 2.5%, which is in keeping with previous population estimates [2].

In our study, we found no clear spatial association relating pharyngeal carriage of *N. meningitidis* to disease, reflecting an incomplete understanding of the dynamics and role of carriage in the transmission of IMD. Other studies, albeit few, have observed an association between higher carriage rates and higher incidence of disease or outbreaks of IMD [13–15], or lower carriage prevalence during a period of low incidence of disease [12]. Hypervirulent meningococcal strains may give rise to higher carriage rates and higher incidence of disease, tempered by meningococcal vaccination contributing to lowered incidence of IMD, particularly in genogroups B and C [12, 32, 33].

Despite finding no clear spatial association as illustrated in the maps relating carriage and disease, we found differences in the spatial distribution of IMD cases and *N. meningitidis* carriage. Environmental, social, and behavioural factors may play a part in disease transmission and acquisition of meningococci [34]. Environmental factors such as dry, windy, and dusty climatic conditions could be correlated with higher carriage prevalence. Exposure to dust may damage the upper respiratory tract mucosa facilitating infection due to increased bacteria load in the nasopharynx explaining the observed higher carriage prevalence in the more remote arid regions of SA [15]. Differences in demographic and socioeconomic characteristics may explain much of the variation we observed between the two regions in terms of the risk of disease and carriage. Metropolitan Adelaide has a younger age distribution than regional SA, and in 2021, it had a higher proportion of people aged over 15 years who had completed their final year of schooling (57% metropolitan vs. 37.6% regional) (https://www.abs.gov.au/statistics/people/population/regional-population-age-and-sex/latest-release).

There is a socioeconomic differential between the two regions, with the highest household income quartile (27.2% metropolitan vs. 17.6% regional) unequally represented. This suggests marked differences in level of education and socioeconomic disadvantage. Behavioural determinants for IMD have been linked to exposure to passive smoke, crowded living conditions, younger age, and low household income [35–37]. The ‘B Part of It’ RCT, from which we drew our study population, found that similar behavioural risk factors were also associated with carriage including current upper respiratory tract infection, cigarette smoking, attending pubs or clubs, and intimate kissing [10]. Higher IMD incidence and carriage prevalence were reported in LGAs in the far west, Outback – North and East, and Anangu Pitjantjatjara, and while we were unable to analyse the data by ethnicity, 21.9%, 6.3%, and 83.6% of Aboriginal and Torres Strait Islander populations in 2016 lived in the regions of Port Augusta (north), Ceduna (far west), and Anangu Pitjantjatjara LGA (Outback – North and East) (https://abs.gov.au/census/find-census-data/quickstats/2016/CED411) in SA, respectively [38]. Recent national statistics also show that Aboriginal and Torres Strait Islander households were larger than non-indigenous households (median of 3.0 people vs. 2.4 people in non-indigenous households) and less likely to report a weekly household income of >AUD$1,000 (16.1% vs. 34.9% in non-indigenous households) [38].

A strength of this research is that it provides a spatial analysis of the distribution of *N. meningitidis* carriage (any and disease-associated) by different genogroups, which we related to IMD case genogroups for the metropolitan area and for the entire state. We used fine-grained spatial analysis that did not compromise confidentiality, as IMD cases were aggregated to postcode centroids, and we reported only aggregated data. Other studies have also used postcodes to identify clusters of IMD cases [17, 22] as postcodes provide a more precise unit for spatial analysis than LGAs. Our spatial analysis accounted for IMD genogroups, a refinement in comparison with other studies that analysed the clustering of IMD cases but in the absence of genogroups [20].

Our study is descriptive; hence, we were not able to infer causal associations between higher carriage density and possible clusters or outbreaks of meningococcal disease. Using place of residence may be a limitation and underestimate the relationship between carriage and disease, as transmission could have occurred at a different location [16]. We did not adjust for clustering by school as the unit of analysis used for mapping the geographical distribution of carriage and IMD was by postcode of residence and by LGA, not by school location. Although our study covered four consecutive years, temporal analysis is not provided as there was unlikely to be significant variation in both carriage and IMD within such a short time period. Despite the decrease in the number of IMD notifications in SA in 2020, we did not exclude the five cases as it was unlikely to have had a significant impact on the analyses. Overall IMD notifications in SA were low in 2020, mirroring a concurrent reduction in influenza cases likely attributable to public health interventions to contain the spread of COVID-19 including the effectiveness of the meningococcal B vaccination programme described earlier [11, 39, 40].

We used an age-specific sub-group of the population to estimate carriage prevalence and to show the spatial distribution, while for IMD cases we used notifications across all age groups. This decision was made because IMD remains, fortunately, rare, and so the sparse data precluded some more refined analyses. However, the distribution of IMD in our study highlighted that adolescents carried a high burden of disease, second only to young children across all notifications during the four-year study period. Understanding *N. meningitidis* carriage and acquisition by high-risk populations such as secondary school students is important in elucidating disease transmission and supporting evidence for implementing a public health response. Future research may include spatial analysis of hypervirulent stains, meningococcal vaccine coverage, and other factors such as environmental and socioeconomic disadvantage that could also help to understand variation in the distribution of *N. meningitidis* carriage and IMD cases.

## Conclusion

Spatial analyses using carriage and IMD genogroups may provide new insights into spatial variation between different genogroups and can help identify geographical areas with higher disease incidence and carriage prevalence. There is a need to better understand the complex environment and host factors which may influence transmission dynamics and carriage prevalence. Individual and area-level social and environmental factors are likely to play a role in the risk of IMD; thus, prevention and control policies should consider these factors in targeting at-risk populations. An improved understanding of the geographical variation of IMD cases may provide opportunities for disease control as well as opportunities for targeted campaigns designed to improve vaccine uptake.

## Data Availability

Data requests can be made to the corresponding first author.
